# Performance Evaluation of ChatGPT-4o in Dermatological Diagnoses Across Fitzpatrick Skin Types

**DOI:** 10.7759/cureus.87368

**Published:** 2025-07-06

**Authors:** Priya Patel Housley, Arthur M Samia, Kiran Motaparthi

**Affiliations:** 1 Department of Dermatology, University of Florida, Gainesville, USA

**Keywords:** artificial intelligence, large language models, melanoma, skin cancer, skin phototype

## Abstract

ChatGPT-4o, a multimodal AI model released in May 2024, offers free image analysis capabilities that patients may access prior to seeking medical care. We evaluated ChatGPT-4o’s diagnostic performance across Fitzpatrick skin types (FSTs) using 324 biopsy-confirmed dermatologic images. The model generated three differential diagnoses and malignancy assessments per image, which were compared to histopathologic ground truths. Results showed significantly lower sensitivity, specificity, and accuracy for melanoma in darker skin tones (FSTs III-VI) compared to lighter tones (FSTs I-II). These findings highlight the potential risk of biased AI performance in underrepresented populations and underscore the need for more inclusive dermatologic datasets.

## Introduction

In May 2024, OpenAI launched ChatGPT-4o, which provides multimodal capabilities and, to date, is more capable than existing models at analyzing dermatoscopic images [1]. As this model is freely available, patients now have greater access to artificial intelligence tools before seeking healthcare [2]. ChatGPT-4o has not been tested for performance in lesion identification and malignancy classification across diverse skin tones [3,4]. Therefore, we evaluated ChatGPT-4o's ability to determine differential diagnoses and assess for malignancy across skin tones utilizing a diverse dataset of images of neoplasms with biopsy-confirmed diagnoses.

## Technical report

**Figure 1:**
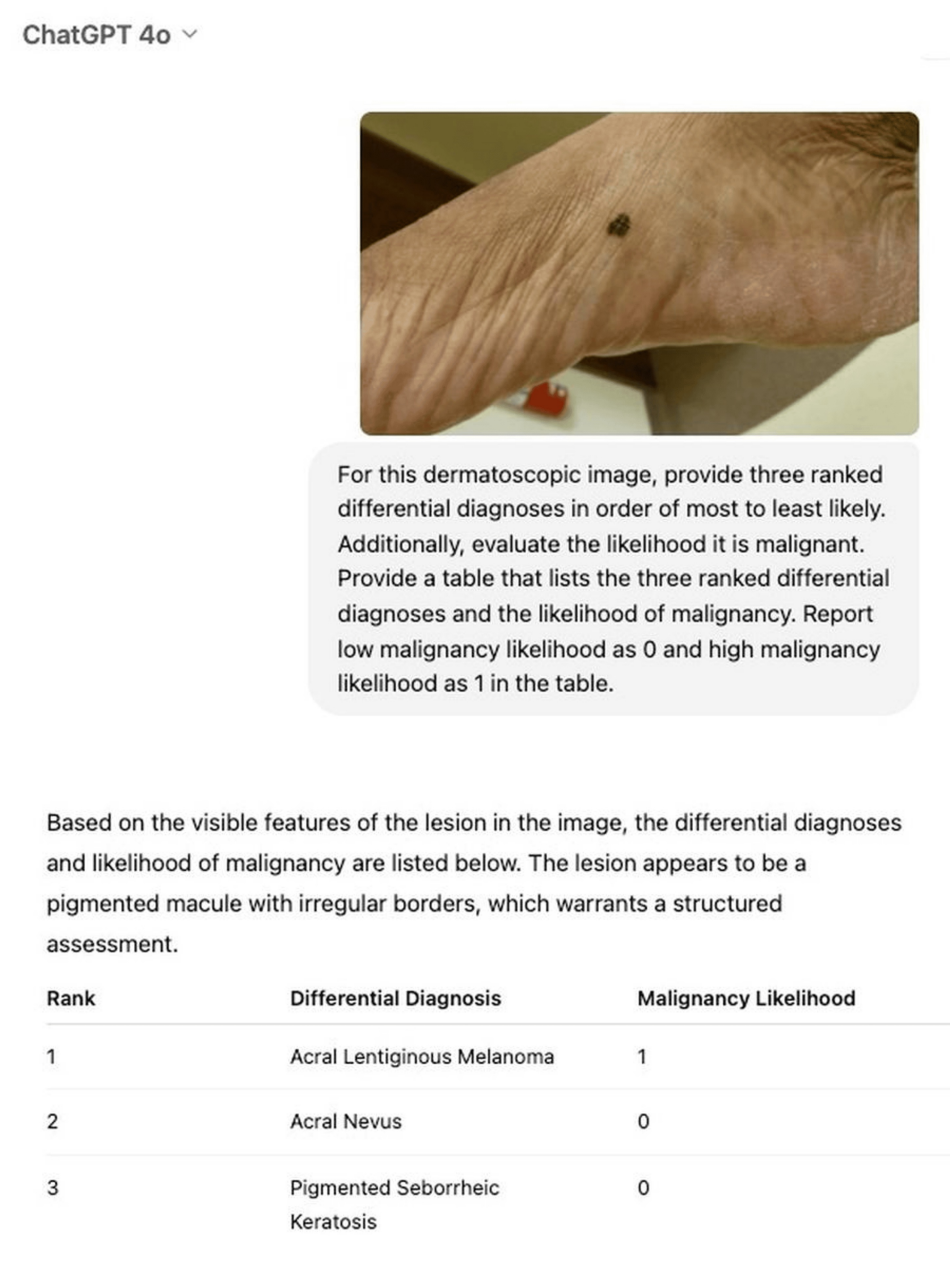
Example exchange with ChatGPT-4o. The model was given a dermatoscopic image of a neoplasm with a biopsy-confirmed diagnosis and was prompted to provide three ranked differential diagnoses and an associated malignancy classification. The responses included a table with the top three diagnoses and corresponding likelihoods of malignancy.

We utilized the Diverse Dermatology Images dataset, which contains diagnosis and malignancy labels based on pathology reports and Fitzpatrick skin type (FST) labels based on in-person clinic evaluations [5]. The labels were verified by two board-certified dermatologists [5]. We selected six lesion categories with both clinical relevance, as determined by a board-certified dermatologist, and high image frequencies from the dataset. The images (n = 324) were used to prompt ChatGPT-4o, as shown in Figure 1, to return three ranked differential diagnoses with malignancy status. We compared the model's output for the top 1 and 3 differentials and malignancy classification to the histopathology labels from the dataset. To study differences in performance among FSTs, we calculated sensitivity, specificity, and accuracy and performed statistical testing using Python 3.1. To explore if FST affected ChatGPT-4o's ability to determine differential diagnoses, we performed Z-tests for two proportions. To evaluate if FST impacted malignancy detection, we conducted Kruskal-Wallis H tests accounting for the non-normal distributions.

**Figure 2:**
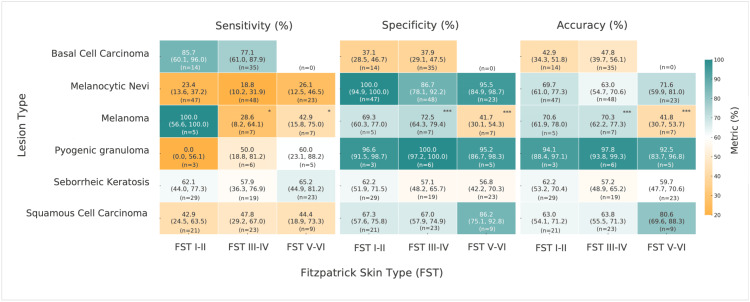
Top 1 differential metrics across lesion types and Fitzpatrick skin types (FSTs) as determined by ChatGPT-4o. This heat map displays values for sensitivity, specificity, and accuracy, followed by the 95% confidence interval determined using the Wilson method, for various lesions stratified by FST groups. Low and high values are displayed using a gradient from orange to teal, respectively. *p ≤ 0.05, ***p ≤ 0.001.

For the top 1 differential lesion identification, sensitivity, specificity, and accuracy values can be found in Figure 2. Notably, for melanoma, FSTs I-II displayed a sensitivity of 100% ± (95%, 57-100%) while FSTs III-IV and V-VI displayed 29% (95%, 8-64%) and 43% ± (95%, 16-75%), respectively. Additionally, FSTs I-II displayed an accuracy of 71% ± (95%, 62-78%), while V-VI displayed 42% ± (95%, 30-54%) for melanoma detection. For melanoma identification in the top 1 differential, Z-test results suggest a statistically significant decrease in sensitivity (FSTs I-II vs II-III, z=2.47, p=0.013; FSTs I-II vs V-VI, z=2.07, p=0.038), specificity (FST I-II vs V-VI, z=3.53, p<0.001; FSTs III-IV vs V-VI, z=4.10, p<0.001), and accuracy (FST I-II vs V-VI, z=3.85, p<0.001; FSTs III-IV vs V-VI, z=3.92, p<0.001) in darker FSTs compared to lighter FSTs.

Additionally, performance in the top 3 differential identification and lesion classification as benign or malignant across FSTs was evaluated. Within the top 3 differentials, ChatGPT-4o demonstrated a lower sensitivity in melanoma detection in darker skin tones (FSTs I-II vs II-III, z=2.58, p=0.01; FSTs I-II vs V-VI, z=2.71, p=0.007). When classifying lesions as benign or malignant, significant differences were not found in dark skin tones.

## Discussion

A previous study elucidated the diagnostic accuracy of an earlier model, ChatGPT-4, in diagnosing melanoma and found gaps in performance [3]. While these researchers utilized the International Skin Imaging Collaboration (ISIC) dataset, we utilized the Diverse Dermatology Images dataset, which was designed to benchmark algorithm performance across diverse skin types [5]. This study suggests that ChatGPT-4o is less sensitive and specific in identifying melanoma in patients with darker skin tones (FSTs III-VI) compared to those with lighter skin tones (FST I-II). We observed this statistically significant disparity when analyzing both the top 1 and top 3 differential diagnoses, which raises concerns about the clinical application of this tool.

As ChatGPT-4o is now publicly available, usage by patients, especially by those of darker skin types, may exacerbate existing healthcare disparities. Compounded with the misconception that people of color do not develop skin cancer, there is a heightened risk of delayed or missed melanoma diagnoses [6]. Therefore, the need for diverse datasets and refined models remains crucial.

Limitations of our study include an uneven image distribution across skin tones and lesion categories; in particular, the dataset did not include images of basal cell carcinoma (BCC) in FSTs V-VI. Additionally, ChatGPT-4o was prompted with solely dermatoscopic images without additional clinical context such as patient history or anatomical location. Therefore, these findings reflect isolated model performance based solely on images. Future work may look to explore performance in a clinical setting after optimizing the model and future prompts for diagnostic equity.

## Conclusions

ChatGPT-4o demonstrates significantly lower sensitivity, specificity, and accuracy for melanoma diagnosis in individuals with darker skin tones. As AI tools become more accessible, these disparities may contribute to delayed or missed diagnoses in vulnerable populations. These findings underscore the urgent need for more representative datasets and model refinement to promote diagnostic equity.
